# Obesity-Related Changes in High-Density Lipoprotein Metabolism and Function

**DOI:** 10.3390/ijms21238985

**Published:** 2020-11-26

**Authors:** Julia T. Stadler, Gunther Marsche

**Affiliations:** Otto Loewi Research Center, Division of Pharmacology, Medical University of Graz, 8010 Graz, Austria

**Keywords:** obesity, HDL-C, HDL subclasses, cholesterol efflux, adiponectin, sphingosine 1-phosphate, bariatric surgery

## Abstract

In obese individuals, atherogenic dyslipidemia is a very common and important factor in the increased risk of cardiovascular disease. Adiposity-associated dyslipidemia is characterized by low high-density lipoprotein cholesterol (HDL-C) levels and an increase in triglyceride-rich lipoproteins. Several factors and mechanisms are involved in lowering HDL-C levels in the obese state and HDL quantity and quality is closely related to adiponectin levels and the bioactive lipid sphingosine-1-phosphate. Recent studies have shown that obesity profoundly alters HDL metabolism, resulting in altered HDL subclass distribution, composition, and function. Importantly, weight loss through gastric bypass surgery and Mediterranean diet, especially when enriched with virgin olive oil, is associated with increased HDL-C levels and significantly improved metrics of HDL function. A thorough understanding of the underlying mechanisms is crucial for a better understanding of the impact of obesity on lipoprotein metabolism and for the development of appropriate therapeutic approaches. The objective of this review article was to summarize the newly identified changes in the metabolism, composition, and function of HDL in obesity and to discuss possible pathophysiological consequences.

## 1. Introduction

The increasing prevalence of obesity in the last decades has become a major health problem worldwide. In Northern America and Europe, in particular, the number of overweight and obese people is ever increasing and is becoming more common in children and adolescents [1]. The causes of obesity are multifactorial, with the most important factors being excess calorie intake and lack of physical activity. Excessive body weight increases the risk of disease development, such as coronary artery disease, hypertension, type-2 diabetes mellitus, and dyslipidemia [2,3,4,5,6]. High levels of triglyceride-rich lipoproteins and low levels of high-density lipoprotein cholesterol (HDL-C) commonly characterize dyslipidemia in obesity. In obesity, not only HDL levels are altered, but an altered HDL distribution pattern and abnormal HDL metabolism have also been observed, which often leads to dysfunction of the HDL particles [7,8,9]. Consequently, the focus has shifted from studying the quantity of HDL to studying the quality of HDL [10]. The current review will focus on HDL metabolism and the pathophysiological changes seen in obesity. Further, we will focus on obesity-induced changes in HDL composition and the concomitant changes of HDL functionality. Another aspect will be the relationship of HDL with the adipokine adiponectin as well as with the bioactive lipid sphingosine-1-phosphate (S1P), whose levels are altered in the state of obesity. We also summarize the effects of weight loss induced by bariatric surgery, Mediterranean diet and pharmacological approaches, which effectively increase HDL-C levels and improve HDL function.

## 2. HDL Metabolism, Structure, and Composition

### 2.1. HDL Metabolism

**Figure 1:**
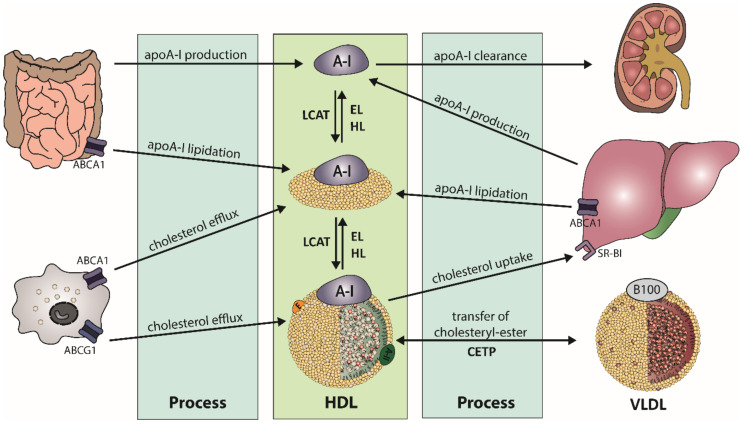
Schematic overview of high-density lipoprotein (HDL) metabolism. Biogenesis of apolipoprotein A-I (apoA-I) takes place in the liver and intestine. After secretion of the lipid-poor apoA-I, it interacts with ATP-binding cassette transporter A1 (ABCA1) to acquire lipids, leading to formation of nascent HDL. The enzyme lecithin-cholesterol-acyl transferase (LCAT) esterifies free cholesterol of nascent HDL to form mature HDL. Cholesteryl-esters are cleared by uptake of the liver by scavenger receptor B1 (SR-BI) or via transfer on triglyceride-rich lipoproteins by cholesteryl-ester transfer protein (CETP), in exchange of triglycerides. Triglyceride-rich HDL is susceptible to hydrolysis by endothelial lipase (EL) or hepatic lipase (HL).

The biogenesis of HDL starts in the liver and the intestine, where apolipoprotein (apo) A-I is synthesized (Figure 1). After secretion, lipid-poor apoA-I interacts with the integral cell membrane protein ATP-binding cassette transporter A1 (ABCA1), which is abundantly expressed by hepatocytes and enterocytes [11]. Through interaction, apoA-I acquires lipids from the cellular lipid pool, generating nascent HDL particles. Additional lipids and apolipoproteins are acquired, which are derived from hydrolysis of triglyceride-rich lipoproteins. This process partly explains the strong inverse relationship of HDL-C and triglyceride levels, often observed in obese subjects [12]. The acquired cholesterol of HDL is further esterified by lecithin-cholesterol-acyl transferase (LCAT), forming mature HDL particles [13]. The reaction takes place at the surface of HDL and requires apoA-I as an activator for LCAT [14]. The generated HDL-associated cholesteryl-esters are partially transferred to apoB-containing lipoproteins by cholesteryl-ester transfer protein (CETP), usually in exchange for triglycerides. Another pathway for clearance of cholesteryl-ester in HDL is the direct uptake by the liver via scavenger receptor class B type 1 (SR-BI) [15]. After interaction of SR-BI with large cholesterol-rich HDL, cholesteryl-esters and free cholesterol are internalized and cholesterol is removed through the bile, while apoA-I dissociates [16,17].

HDL is enriched in triglycerides through the activity of CETP, generating HDL particles that are more susceptible to lipolysis by endothelial lipase (EL) or hepatic lipase (HL). Substrates for lipolysis are mainly phospholipids (EL) or phospholipids and triglycerides (HL), but with different specificity for phospholipids [18]. The lipolysis of triglycerides leads to the formation of smaller HDL particles, which are susceptible to faster catabolism. Another important key player of HDL metabolism is the phospholipid transfer protein (PLTP), which transfers phospholipids between HDL particles and lipids between triglyceride-rich lipoproteins and HDL [19]. Many apolipoproteins, lipid transfer proteins, enzymes, cell surface receptors, and cellular lipid transporters are involved in the regulation of HDL metabolism and partly determine levels of plasma HDL-C. This complex metabolism produces HDL particles of varying size, density, and composition. Therefore, plasma HDL-C concentrations are not a good parameter to reflect functional properties of HDL, such as HDL-mediated reverse cholesterol transport or anti-oxidative or anti-inflammatory properties.

### 2.2. HDL Structure and Composition

Plasma levels of HDL-C have been associated with cardiovascular diseases for decades [20,21,22]. However, it is becoming widely accepted that it is not the quantity but the quality of HDL that is important, as HDL performs different functions depending on the protein and lipid composition [23,24,25]. ApoA-I is the most prevalent protein component of HDL, accounting for approximately 70% of the total protein [26]. ApoA-I has a variety of functions, such as activation of LCAT, interaction with cellular receptors, and anti-atherogenic activities [27,28,29]. ApoA-II is the second major apolipoprotein in HDL and presents about 15–20% of the total protein component [30]. The remaining 10–15% of HDL protein mass comprises minor proteins, including apoA-IV, ApoCs, which are important enzyme regulators, apoD, apoE, apoF, apoH, apoJ, ApoL-I, and apoM, and several enzymes. Paraoxonase 1 (PON1) is almost exclusively associated with HDL and has been shown to exert anti-inflammatory and anti-oxidative properties [31]. Other enzymes associated with HDL are LCAT and the platelet-activating factor acetyl hydrolase. The phospholipid transfer protein and CETP have a lipid transfer activity and are important in lipoprotein metabolism. Remarkably, it is not cholesterol that predominates the HDL lipidome, but phospholipids. Taken together, phospholipids and sphingolipids account for 40–60% of total lipids, while cholesteryl-ester (30–40%), triglycerides (5–12%), and free cholesterol (5–10%) are less abundant [23]. Similar to functions of HDL-associated proteins, HDL lipids also accomplish distinct structural functions. The lipid surface monolayer is constituted of phospholipids, while cholesteryl-ester and triglycerides form the hydrophobic core. In total, more than 200 lipids and 80 proteins are carried by different HDL subclasses, with individual HDL particles carrying only a few other proteins besides apoA-I [32,33,34].

### 2.3. HDL Subclasses

Multiple subclasses of HDL exist, depending on its stage of maturation, site of origin, and its protein and lipid composition. Thus, HDL particles are highly heterogeneous in their size, shape, structure, and density (Table 1). Pre-β HDL is structurally the simplest form of HDL. These particles consist of one or two apoA-I molecules with a phospholipid layer and a trace amount of cholesterol. These particles are discoidal shaped with a diameter of approximately 9.6 nm and a thickness of 4.7 nm [35]. Pre-β HDL particles rapidly take up cholesterol and phospholipids, which convert them into larger HDL subclasses. Therefore, pre-β HDL only accounts for about 5% of HDL in the circulation [36]. Because of their function to avidly absorb cholesterol and phospholipids, pre-β HDL particles are thought to be a major factor in preventing atherosclerotic plaque formation. Importantly, higher serum cholesterol efflux capacity is related to plasma concentrations of pre-β HDL [37]. HDL3 particles have a smaller diameter (7.5 nm) and are enriched with proteins, while HDL2 particles are larger (10 nm) and lipid rich. Most abundant apolipoproteins are apoA-I and apoA-II in both subclasses; however, apoA-II is more present in HDL3. Interestingly, the HDL-associated enzyme PON1, which has anti-oxidative and anti-inflammatory properties [31], has been shown to be more frequently associated with HDL3. This higher abundance of PON1 on HDL3 could partly explain the higher anti-oxidative capacity of the smaller HDL particles [29,38]. HDL2 and HDL3 further show differences in lipid composition. Sphingolipids are, in general, less abundant in the HDL3 subclass, affecting surface lipid fluidity, whereas the bioactive lipid sphingosine-1-phosphate (S1P) is predominantly associated with HDL3 [23]. In line, the abundance of apoM, which specifically anchors S1P to HDL particle, shows higher abundance in HDL3 [38]. S1P maintains vascular integrity and mediates multiple effects of HDL on endothelial cells [39]. The functions of HDL to induce vasorelaxation as well as promoting barrier function have been attributed to signaling of S1P [40,41]. Taken together, it seems that smaller subclasses of HDL have a greater protective potential than larger particles [29].

### 2.4. Important Functions of HDL

One of the main functions of HDL is its ability to promote reverse cholesterol transport, the uptake of excess cholesterol from peripheral cells, and the transport to the liver for excretion. This process is considered as the major antiatherogenic effect of HDL [42].

The reverse cholesterol transport starts with the secretion of lipid-poor apoA-I, which is released from liver or intestine into the plasma to circulate to peripheral cells from which excess cholesterol is removed, forming nascent HDL. A key role in the reverse cholesterol transport is the interaction of apoA-I with ABCA1 [43]. Studies have shown that ABCA1 preferentially lipidates small HDL, specifically apoA-I, to form nascent HDL, while ATP-binding cassette subfamily G member 1 (ABCG1) stimulates cholesterol efflux to mature HDL and not to lipid-poor apoA-I [44,45]. Cholesterol efflux includes the passive diffusion of cholesterol from cells as well as the active cellular cholesterol transfer by ABCA1, ABCG1, and SR-BI [46,47,48]. The absorbed cholesterol is esterified by LCAT and mature HDL is formed. HDL-associated cholesteryl-ester is partially transferred to triglyceride-rich lipoproteins by CETP and further cleared by hepatic clearance through the low-density lipoprotein (LDL) receptor or taken up together with free cholesterol by the hepatic receptor SR-BI. Therefore, the transfer of cholesterol from peripheral cells to the liver involves two routes: (1) the direct uptake via SR-BI and (2) indirect by HDL-LDL/very low-density lipoprotein (VLDL) interaction [42]. In the liver, the cholesteryl-esters are hydrolyzed, and free cholesterol is either transported by ABCG5 and ABCG8 into the bile for excretion into feces or converted into bile acids or reused for VLDL production.

This process of HDL-mediated cholesterol efflux has been of expanded research interest in recent years. A number of different cell-based assays have been developed, to measure the ability of HDL to promote cholesterol efflux, the first step of reverse cholesterol transport. In the most established assay, a mouse macrophage cell line (J774) was employed [49]. Cells are enriched with radioactively or fluorescently labeled cholesterol and cyclic adenosine monophosphate to upregulate expression of ABCA1. For these assays, isolated HDL or apoB-depleted serum from patients is added to cell medium and the proportion between labeled cholesterol in the supernatant and in the cells is calculated.

Besides the ability of HDL to promote cholesterol efflux, there is increasing evidence that HDL-mediated antiatherogenic actions toward the endothelium have physiological relevance [50,51,52,53].

The beneficial properties of HDL on the endothelium include vasodilatory activity, primarily through stimulation of nitric oxide (NO) release from endothelial cells [40,54], and also the production of prostacyclin [55,56]. The initial step for the activation of NO production involves binding of HDL to SR-BI on the endothelium. Subsequent intracellular events are mediated by endothelial protein kinase B and intracellular Ca^2+^ mobilization, increase in intracellular ceramide levels, and the phosphorylation of the endothelial NO-synthase, leading to NO release [40,57,58,59]. HDL reduces the activity of the nicotinamide adenine dinucleotide phosphate (NADPH) oxidase in the endothelium, which reduces the cellular production of superoxide, an inactivator of NO, thereby increasing NO bioavailability [60]. Vasodilatory actions of HDL further include cholesterol efflux of cholesterol and 7-oxysterols, mediated by ABCG1, which improves formation of active endothelial NO-synthase dimers, resulting in decreased production of reactive oxygen species [61].

Another anti-atherogenic function of HDL is its ant-oxidative activity by protecting LDL from oxidative damage induced by free radicals, thus reducing its atherogenicity. ApoA-I, the major protein component of HDL may play a central role in HDL-mediated anti-oxidative activity, by reduction of lipid hydroperoxides through methionine residues [62,63]. In addition, HDL-associated PON1 was shown to decrease lipid peroxidation of LDL and HDL through a specific cysteine residue [64]. Other apolipoprotein components and HDL-associated enzymes, such as apoA-II, apoE, apoJ, lipoprotein-associated phospholipase A2, and LCAT, may further contribute to the anti-oxidative properties [29,65,66]. HDL-associated lipophilic antioxidants such as tocopherols seem to make a small contribution to the antioxidant properties of HDL [67].

Additionally, to the number of anti-oxidative effects, HDL further possesses anti-inflammatory properties. In vitro experiments have shown that HDL inhibits transmigration of monocytes [68] and inhibits cytokine-induced expression of vascular cell adhesion molecule, intercellular cell adhesion molecule, and E-selectin expression [69,70]. By modulation of the nuclear factor κB and the peroxisome proliferator-activated receptor gamma, HDL further inhibits the production of pro-inflammatory cytokines [71]. Due to these capabilities, HDL reduces the recruitment of lymphocytes, monocytes, and basophils to the vascular endothelium, thereby decelerating downstream events of inflammatory response.

## 3. HDL-C-Raising Therapies and Cardiovascular Outcome

The cholesterol component of HDL has been shown to be inversely associated with the risk of coronary heart disease (CHD) and is a key component of predicting cardiovascular risk in the general population [12]. The Framingham Heart Study was the first study to observe the strong association between HDL-C and CHD and, therefore, served as the basis for the hypothesis that HDL, as the “good” cholesterol, might hold protective properties against CHD [72]. However, more recent data clearly indicate that the association between HDL-C concentration and all-cause mortality is U-shaped, and both extremely high and low HDL-C concentrations are associated with an increase in mortality [73]. This leads to considerable uncertainty about the potential benefit of increasing HDL-C and may reflect or explain the disappointing results of recent clinical studies on a number of therapeutic interventions aimed at increasing HDL-C levels, such as CETP inhibitors [74,75,76]. Given the heterogeneity of HDL particles in terms of structure, size, lipidomic/proteomic composition, and metabolism, HDL-C values are only a snapshot of the steady-state cholesterol pool. HDL-C values provide no direct information on the rate of cholesterol efflux from vascular macrophages in liver, which is influenced by many factors beyond the mass of HDL-C alone. Furthermore, the circulating HDL-C concentrations do not provide information about the anti-inflammatory, anti-oxidant, anti-thrombotic, and endothelial function-promoting activities of HDL [77]. Therefore, considerable interest has recently focused on approaches to influence the biological functions of HDL in the search for new cardioprotective therapies [78,79,80,81,82,83,84]. This is based on new findings that underline the importance of HDL functionality [85,86,87], which has led to ongoing efforts to develop new risk markers and therapeutics that focus on HDL quality rather than quantity.

## 4. Obesity Alters HDL-C Levels

Obesity is commonly accompanied by low HDL-C levels and an increase in triglyceride-rich lipoproteins [88], which is often termed as atherogenic dyslipidemia. Characteristic for this dyslipidemia is a decreased clearance of triglyceride-rich lipoproteins, which is caused by a relative lack of insulin-sensitive lipoprotein lipase [89,90,91]. Lipoprotein lipase hydrolyzes triglycerides of chylomicrons and VLDL, leading to shrinkage of the particles and transfer of surface phospholipids and apolipoproteins to HDL, thus increasing HDL size. During obesity, the response of lipoprotein lipase activity to glucose stimulation has been shown to be reduced [92], representing one potential factor contributing to the decrease of HDL-C in obesity.

**Figure 2:**
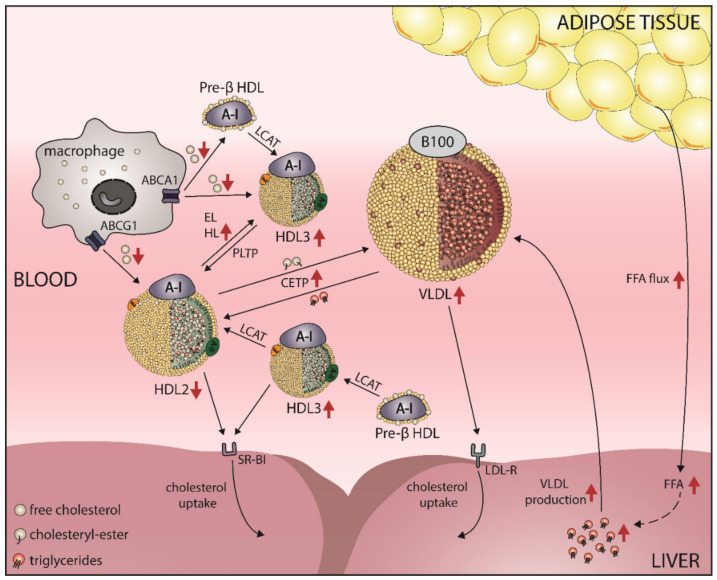
Proposed mechanisms involved in the obesity-induced shift in HDL subclass distribution. Pre-β HDL is rapidly lapidated, leading to the formation of HDL3. The ingested free cholesterol is esterified by lecithin-cholesterol-acyl transferase (LCAT), which leads to maturation of HDL particles. Phospholipid transfer protein (PLTP) transfers phospholipids onto HDL, whereas HDL-associated cholesteryl-esters are transferred to very low-density lipoproteins (VLDL) in exchange of triglycerides by cholesteryl-ester transfer protein (CETP). Pre-β HDL and small HDL3 remove excess cholesterol via ABCA1, while HDL2 removes cholesterol via ABCG1. Endothelial lipase (EL) and hepatic lipase (HL) hydrolyze HDL-associated triglycerides and phospholipids. Cholesteryl-esters of HDL are transported back to the liver by HDL via scavenger receptor B1 (SR-BI) or by VLDL via the LDL receptor. In obesity (indicated with red arrows), there is an increased flux of free fatty acids (FFA) from adipocytes to the liver, which leads to accumulation of triglycerides and an increased secretion of VLDL, resulting in elevated transfer of triglycerides to HDL via CETP. Triglyceride-rich HDL is rapidly hydrolyzed via HL, which shows increased activity, resulting in formation of smaller HDL particles. The ability of HDL to promote cholesterol efflux from lipid-loaded cells is reduced in obesity.

The increase of triglyceride-rich lipoproteins is a causal factor for low HDL-C levels in obesity. The increase in the release of free fatty acids from the adipocytes caused by obesity increases their uptake by the liver, resulting in liver accumulation and enhanced production of VLDL and its release into the bloodstream (Figure 2) [93]. This increase of acceptor lipoproteins further stimulates the transfer of triglycerides on HDL in exchange for cholesteryl-esters mediated by CETP [94]. During this process, HDL is enriched in triglycerides and represents a better substrate for hepatic lipase and is hydrolyzed more rapidly [95]. In obese insulin-resistant subjects, HDL is enriched in triglycerides and the activity of hepatic lipase is increased [96,97,98]. Hydrolysis of triglyceride-rich HDL further leads to the formation of smaller HDL3 particles, which are susceptible to faster catabolism [99]. Interestingly, even when fasting plasma triglyceride levels are at a normal level, obese patients often display low HDL-C levels, suggesting further mechanisms leading to HDL-C lowering in obesity.

Another characteristic of obesity is an imbalance of adipokines, including leptin. Leptin is mainly produced by adipocytes and is elevated in overweight and obese individuals [100,101]. Interestingly, a study in children showed that plasma leptin levels correlated with HDL-C [102]. Further, a correlation between leptin and HDL-associated triglycerides and with HDL particle size has been reported in adults [103]. In vivo experiments in leptin-deficient (ob/ob) mice suggest that leptin upregulates hepatic SR-BI and thereby influences levels of HDL-C [104].

In the state of obesity, increased CETP levels are correlated with leptin levels [105], in line with the fact that adipose tissue is one of the major sources of CETP expression [106]. Therefore, the obesity-associated increase in CETP production is thought to affect HDL-C levels [107].

Another molecule, secreted from adipose tissue, which may have a direct impact on HDL metabolism, is the adipokine adiponectin. Studies have shown that levels of adiponectin, which are reduced in the state of obesity, are directly correlated with plasma HDL-C levels [108,109,110,111,112]. Furthermore, an intervention study showed that levels of adiponectin as well as of HDL-C are increasing after weight loss and that this improvement was independent of changes in insulin sensitivity and fat mass [113]. The relationship of HDL with adiponectin will be discussed in Section 5.3 in more detail.

As mentioned above, the activity of hepatic lipase is increased in obesity and insulin resistance [96,97,98,114,115], leading to faster clearance of triglyceride-rich HDL [116], which is produced by CETP-mediated transfer. The triglyceride-enriched HDL is a more susceptible substrate for hepatic lipase and, therefore, undergoes rapid hydrolysis [99,117]. The mechanisms underlying the increase in hepatic lipase activity in obese states are not yet understood, but it appears that hepatic insulin resistance plays an important role [118]. However, further studies are needed to clarify the link between HDL metabolism and hepatic lipase expression in obesity and insulin resistance.

Another lipase, which may affect HDL-C levels in obesity or insulin resistance is endothelial lipase. Experiments with rodents already revealed the impact of endothelial lipase on HDL metabolism: Inhibition or genetic deletion of endothelial lipase resulted in elevated levels of HDL-C by reduction of catabolism rate [119,120,121], while overexpression of endothelial lipase caused a reduction of HDL-C by increased catabolism rate [119,122,123]. Human studies further have shown that some rare genetic variants in the endothelial lipase gene are linked with high HDL-C levels and that they are correlated to levels of plasma endothelial lipase mass [124,125]. In obesity, levels of endothelial lipase have been shown to be significantly elevated, proposing an upregulation of endothelial lipase during obese states, which may contribute to the reduced HDL-C levels [124]. Obesity is characterized by low-grade inflammation, leading to infiltration of immune cells into adipose tissue [126,127]. The obesity-induced inflammation may decrease HDL-C levels by upregulation of endothelial lipase. However, the significance of endothelial lipase on low levels of HDL-C in the obese state further needs to be investigated.

Another factor affecting HDL-C is cholesterol released by adipocytes. In humans, adipose tissue is a major site for cholesterol storage and contains up to 25% of total body cholesterol in normal-weight subjects and approximately half of it in obese states [128,129]. In adipose tissue, nearly all of the cholesterol is stored in the unesterified form, as free cholesterol, which makes adipocytes unique among cells [129,130,131,132]. It is well reported that adipocytes express the major cholesterol transporters ABCA1 and SR-BI as well as ABCG1, but in a much lesser extent [133]. Adipocytes are promoting cholesterol transfer to HDL via ABCA1 and SR-BI, representing a direct factor for modulation of HDL-C levels. Importantly, Zhang et al. demonstrated that lack of adipose ABCA1 resulted in reduced levels of HDL-C and caused a backlog of cholesterol within adipose tissue [134]. Further, they showed that adipocyte inflammation, which is a hallmark of central obesity, downregulates ABCA1 and SR-BI expression and impairs cholesterol efflux from adipocytes to HDL. Therefore, their results suggest a direct impact of adipose tissue on modulation of HDL-C and that obesity-induced inflammation of adipocytes may result in impaired cholesterol efflux to HDL, contributing to reduced HDL-C levels.

Concluding, several factors and mechanisms are involved in the reduction of HDL-C levels in the obese state, but further research on these mechanisms is of importance to find novel treatment strategies improving HDL quality and quantity.

## 5. Obesity, HDL, and Cardiovascular Risk

Obesity is one of the major risk factors for cardiovascular disease, which is associated with atherogenic dyslipidemia. These alterations in plasma lipid and lipoprotein levels contribute to the manifestation of such a severe morbidity.

### 5.1. Obesity Leads to a Shift in HDL Subclass Distribution

As described above, plasma HDL-C levels do not adequately reflect protective functions of HDL and greater protective potential is attributed to the smaller, more dense HDL particles. Recent studies of Woudberg et al. assessed HDL subclass distribution in normal-weight and obese white and black South African women. In obese study participants, a shift from large HDL toward increased levels of intermediate and small HDL subclasses was seen, whereby the effect was more pronounced in white women [135]. In a 5.5-year follow-up study they showed that the shifts in HDL subclass distribution were related to increasing central adiposity, suggesting a link between body fat distribution and lipid metabolism [8]. Based on the observed changes in HDL subclass distribution in obese individuals, Woudberg et al. explored the effect of exercise training on HDL subfractions. Interestingly, 12 weeks of exercise intervention altered the distribution of small HDL in obese women [136].

In adolescents suffering from type 2 diabetes mellitus, Davidson et al. determined the risk factors associated with the depletion of large HDL particles and simultaneous accumulation of small particles [137]. The authors investigated the distribution of HDL subclasses of individuals who differed in body mass index and insulin sensitivity and found that obesity is the major risk factor linked to the altered HDL subclasses. An increased CETP-mediated transfer of triglycerides on HDL and the subsequent hydrolysis of triglyceride-enriched HDL by hepatic lipase appeared to be the mechanism underlying the shift of large HDL to small and dense HDL particles [137].

### 5.2. Obesity Affects HDL Function

It is known that HDL functionality is severely impaired in certain diseases and HDL may even have inflammatory or pro-atherogenic properties. This was clearly demonstrated in HDL from patients suffering from chronic kidney disease [138,139], diabetes [140], cardiovascular disease [86], liver disease [141], psoriasis [142], or even atopic dermatitis [143] and allergic rhinitis [144]. Obesity-associated complications, such as inflammation or diabetes, have been shown to render HDL dysfunctional. HDL isolated from type 2 diabetes patients did not reduce endothelial oxidant stress and did not improve endothelium-dependent vasodilatation when compared to HDL isolated from healthy subjects [145]. Vasodilatory activity of HDL has been shown to be inversely correlated with triglyceride content of HDL, which is elevated in obesity [146]. A reduction of the overall capacity of HDL to promote cholesterol efflux from fibroblasts in obese, compared to lean, normal-weight, subjects was reported [147]. Of particular interest, cholesterol efflux capacity appears to be significantly inversely correlated with the body mass index [148,149]. Since cholesterol efflux capacity is the main metric of HDL function and has strong inverse association with coronary artery disease [85,150,151], the reduction of efflux capacity in obesity may have a crucial impact on the development of cardiovascular disease.

### 5.3. Adiponectin and HDL

It has been well reported that plasma HDL-C concentrations show a strong correlation with levels of adiponectin, independent of body mass index, distribution of body fat, and insulin sensitivity [108,109,110,111,112]. Adiponectin is mainly secreted by adipocytes, shows anti-atherogenic properties, and modulates glucose metabolism [152,153]. Studies with mice overexpressing or lacking adiponectin as well as in vitro studies suggest a causal relationship with HDL-C levels.

**Figure 3:**
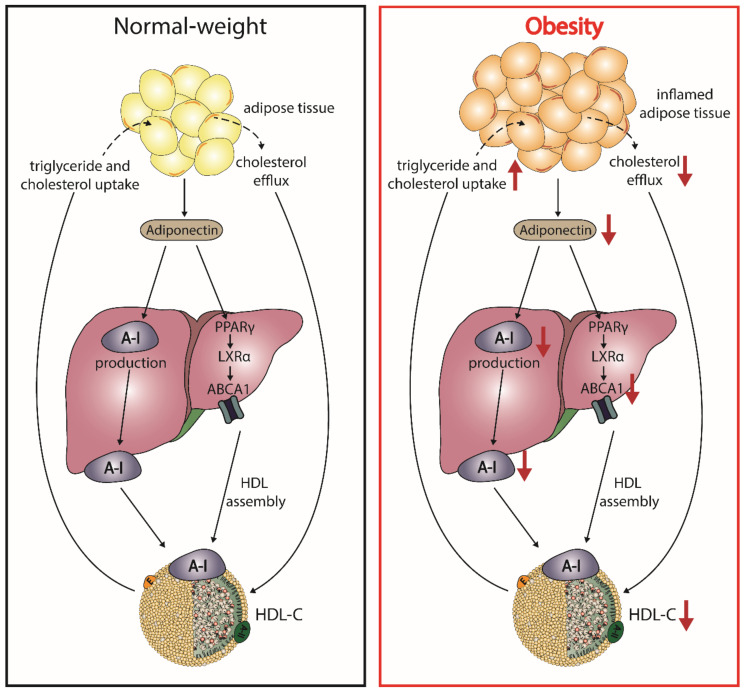
Postulated effects of obesity on adiponectin and HDL metabolism. In normal-weight subjects, adipocytes produce adiponectin, which enhances expression of the ATP-binding cassette transporter A1 (ABCA1) through activation of peroxisome proliferator-activated receptor gamma (PPARγ) and liver X receptor alpha (LXRα), leading to HDL assembly. Further, adiponectin increases the hepatic production of apoA-I. During the state of obesity, adipocytes manifest several altered properties, which play a role in the reduction of HDL-C. Increased inflammation and fat accumulation in the adipocytes reduces the production of adiponectin and impairs cholesterol flux to HDL. The reduction of adiponectin downregulates apoA-I production and ABCA1 expression in hepatocytes, thus reducing HDL assembly.

Adiponectin increases the production of apoA-I as well as hepatic ABCA1, which increases HDL-C levels (Figure 3) [154,155]. The enhanced expression of ABCA1 has been suggested by activation of liver X receptor alpha and peroxisome proliferator-activated receptor gamma [156,157,158]. Plasma levels of adiponectin show a negative correlation with fractional catabolic rate of apoA-I in individuals with metabolic syndrome and control subjects [159]. Besides ABCA1, adiponectin upregulates ABCG1 expression, increases cholesterol efflux capacity, and efficiently promotes lipidation of apoA-I, leading to formation of nascent HDL [160].

Adiponectin has been consistently reported to be associated with cholesterol efflux capacity of HDL [148,161,162]. Other studies have shown an inverse association of hepatic lipase with serum adiponectin levels [163,164]. Adiponectin might inhibit the hepatic lipase-mediated hydrolysis of triglycerides and phospholipids of HDL2 particles. This is in line with studies showing an association of adiponectin with HDL particle size and HDL2 [165,166,167,168]. However, further studies are needed to prove causality. Another further mechanism suggested that adiponectin increases lipoprotein lipase activity, thereby accelerating clearance of triglyceride-rich particles. This, in turn, would lead to less exchange of triglycerides and cholesteryl-ester by CETP and, thus, to cholesteryl-ester-enriched HDL2 particles. A positive correlation between circulating adiponectin and post-heparin lipoprotein lipase activity has been reported [169,170], but causality has to be proven to draw firm conclusions. Low-grade inflammation and fat accumulation cause a dysregulated adipokine production [171,172] and markedly reduce adiponectin levels [173,174]. Therefore, low adiponectin observed in obesity levels may explain, at least in part, the shift of large HDL to small HDL particles.

### 5.4. Obesity and HDL-Associated Sphingosine-1-Phosphate (S1P)

Of particular importance, the complete sphingolipid metabolism is altered in obesity [175]. In obese individuals the levels of ceramides, sphingosine, sphinganine, and S1P are increased in adipocytes when compared to lean controls [175]. The bioactive lipid S1P is mainly carried via apoM anchored to HDL (about 65%) and to a lesser extent via albumin (about 25%) or LDL/VLDL (about 10%) [39]. The half-life of S1P is prolonged when it is associated with HDL, when compared with albumin-associated S1P [176]. S1P is a member of the sphingolipid family, a large group of molecules with a wide range of physiological functions. S1P in the circulation is mainly derived from erythrocytes, vascular endothelial cells, and platelets [177,178]. S1P activates five different G protein-coupled receptors, termed S1P receptors 1–5 (S1PR1–5), in an autocrine or paracrine manner [179].

Kowalski et al. observed that levels of S1P are elevated in plasma of obese humans and rodents and that the levels correlate with metabolic abnormalities such as adiposity and markers of insulin resistance [180]. However, this increase of S1P could not be confirmed in a study comparing levels between overweight and lean adolescents [181]. More recently, the group of Green et al. analyzed the liver metabolome of mice after caloric restriction and revealed that caloric restriction had an impact on S1P signaling [182]. The authors observed that as a response to caloric restriction, liver expression of S1P was significantly increased. S1P levels were negatively associated with decreasing body mass, leptin, and insulin-like growth factor-1. Another study investigated the role of S1P/S1PR1 signaling in the regulation of energy homeostasis in rodents [183]. The authors showed that S1PR is highly expressed in the hypothalamus and that a fasting period of 12 h could reduce S1PR level, whereas refeeding restored the protein levels of the receptor in the hypothalamus. Altogether, their results indicated that the S1P/S1PR1 axis plays a critical role in energy balance and represents a potential target for treatment of obesity. The potential role of S1P signaling in energy metabolism was strengthened by Christoffersen et al., showing that lack of apoM in mice increases the amount of brown adipose tissue and that the turnover of fat is increased, resulting in low white adipose tissue mass and low body weight [184]. These effects of apoM knockout suggest that pharmacological modulation of S1PRs may be a promising approach for the treatment of obesity and associated diseases in the future [185].

Noteworthily, only a small number of studies investigated plasma levels of S1P in obese or overweight human subjects. While obesity is associated with a shift from large HDL to small and dense HDL, S1P has to be increasingly transported with alternative chaperones, reducing the effectiveness of S1P [8]. In line with this hypothesis, Frej et al. showed that a shift in apoM/S1P between HDL particles in women was associated with impaired anti-inflammatory effects of the apoM/S1P complex [186].

## 6. Bariatric Surgery Improves HDL Levels and Function

Bariatric surgery has been demonstrated as the most effective intervention for patients with severe obesity, which induces sustained long-term weight reduction associated with decreased obesity-associated comorbidities and cardiovascular mortality [187,188,189,190]. The standard bariatric surgeries are Roux-en-Y gastric bypass (RYGB), where most of the stomach is bypassed, creating a small gastric pouch; whereas sleeve gastrectomy resects the gastric fundus and most of the gastric body [191]. RYGB surgeries resulted in significant improvements of plasma lipid levels, decreased risk of cardiovascular disease, and overall mortality [192,193,194,195]. Further, after RYGB, levels of circulating adiponectin increased, insulin sensitivity improved, and blood pressure levels were reduced [196,197,198].

**Figure 4:**
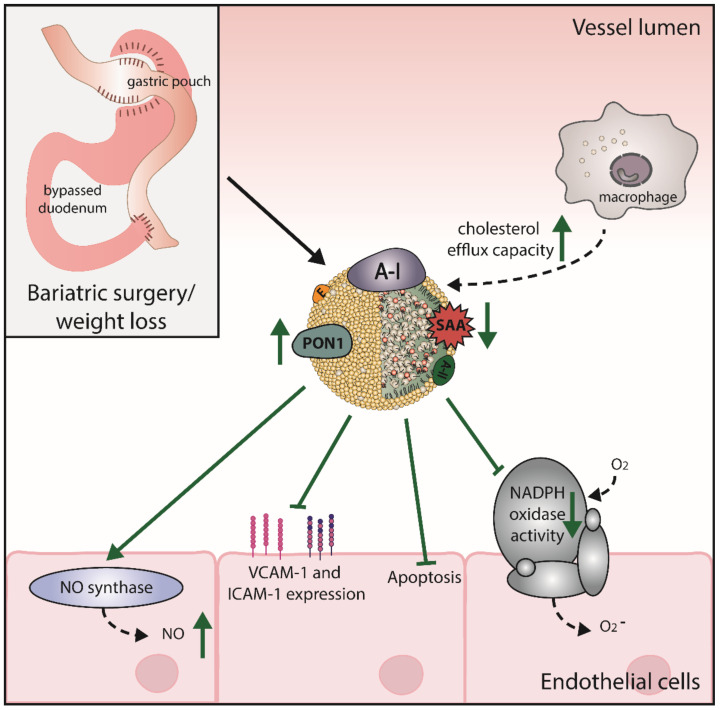
Proposed effects of bariatric surgery on metrics of HDL function. After surgical procedure, HDL shows increased cholesterol efflux capacity, improved paraoxonase 1 (PON1), and reduced levels of serum amyloid A (SAA). Further, HDL of patients after bariatric surgery inhibits expression of the vascular adhesion molecule (VCAM-1) and intracellular adhesion molecule (ICAM-1), improves endothelial anti-apoptotic properties, and reduces nicotinamide adenine dinucleotide phosphate (NADPH) oxidase activity. Bariatric surgery also improved HDL-mediated production of nitric oxide (NO) and, thus, improved endothelial function.

Of particular interest is that the plasma levels of HDL-C after bariatric surgery were remarkably improved compared to the preoperative values and compared to people who only received medical therapy for weight loss [195,199,200,201,202]. In the Surgical Treatment and Medications Potentially Eradicate Diabetes Efficiently (STAMPEDE) clinical trial, obese patients with type 2 diabetes mellitus were randomly assigned to receive intensive medical therapy alone or in combination with RYGB or sleeve gastrectomy. Five years after surgical procedures, the levels of HDL-C were increased by 32%, 30%, and 7% in the RYGB, sleeve gastrectomy, and medical therapy alone groups, respectively [201]. In a substudy, Lorkowski et al. investigated serum HDL function, by determining the apoA-I exchange rate and cholesterol efflux capacity in the STAMPEDE study. The apoA-I exchange rate is determined by adding labeled apoA-I to serum samples and recording labeled apoA-I incorporation into serum HDL [203]. This apoA-I exchange rate has been linked with risk of major adverse cardiovascular events [203]. HDL in both RYGB and sleeve gastrectomy groups showed improved functionality, by increased apoA-I exchange rate after one and five years compared to baseline. Moreover, also cholesterol efflux capacity after five years was improved when compared to pre-operative samples (Figure 4). Improvement of cholesterol efflux capacity appears to depend on the procedure, with an improvement only with sleeve gastrectomy, but not with RYGB at six months after surgery [204]. However, after 12 months both operations resulted in improved cholesterol efflux capacity [204].

In addition, other metrics of HDL function were assessed in morbidly obese patients after bariatric procedure. Six months after surgery, the antioxidant potential of HDL was increased, accompanied by an increase in PON1 protein levels. Further, alterations in the distribution of HDL subpopulations with a shift toward more mature HDL as well as an increase in apoA-I/apoE ratio was found [205].

Laparoscopic adjustable gastric banding is another type of weight-loss surgery, which is minimally invasive and associated with low rates of associated complications and mortality rates [206]. Recently, the impact of laparoscopic adjustable gastric banding on HDL subclass distribution was studied [207]. The authors observed an increase in large HDL and intermediate HDL subclasses and a decrease of the small HDL subfraction [207]. Similar to this, another study observed an increase in the large HDL subfractions after laparoscopic adjustable gastric banding and a reduction of HDL-associated pro-inflammatory serum amyloid A [208].

Another study evaluated whether RYGB restores protective properties of HDL and reverses the obesity-induced endothelial dysfunction [209]. In a rat model of RYGB as well as in human samples, endothelium protective activities of HDL were improved and associated with increased plasma levels of the gut hormone glucagon-like peptide-1 and bile acids. HDL isolated from patients after RYGB led to restored endothelial nitric oxide synthase, increased nitric oxide release and, in parallel, a reduction of endothelial nicotinamide adenine dinucleotide phosphate oxidase, and decrease in endothelial apoptosis and vascular adhesion molecule expression. Moreover, the ability of HDL to induce cholesterol efflux from macrophages as well as PON1 activity was enhanced. Interestingly, 12 weeks after RYGB, the properties of HDL were improved to levels of healthy subjects, although the patients were still obese [209]. A recently published study confirmed the improvement of cholesterol efflux capacity and PON1 activity 12 months after RYGB and observed an association of miR-222 and miR-223, both reported to play an important role in the pathophysiology of obesity [210,211], with markers of HDL function [212].

Altogether, the current state of research suggests that the marked increase in HDL quality and quantity observed after bariatric surgery is likely linked to reduction of obesity-related comorbidities and cardiovascular mortality.

## 7. Effects of Pharmacological Anti-Obesity Interventions on HDL Levels and Function

Changes in dietary and physical lifestyle have been shown to result in a limited reduction in bodyweight (3–10%) and that most people regained weight again [213]. Therefore, besides bariatric surgery, complementary treatments with anti-obesity drugs are a strategy to achieve permanent weight loss in pathologically obese individuals. In 1959, the first anti-obesity drug, termed phentermine was approved by the United States Food and Drug Administration Nowadays, a number of pharmacotherapies have become available to treat obesity.

Phentermine belongs to the group of sympathomimetics and is the most commonly prescribed anti-obesity drug in the USA [214]. Twelve weeks of administration of phentermine reduced body weight and decreased levels of total cholesterol in Korean obese subjects [215].

A combination therapy of phentermine with topiramate has been shown to induce greater weight loss than either drug alone and showed fewer occurrence of side effects [216]. Administration of phentermine and topiramate in overweight and obese patients with dyslipidemia showed improvements in HDL-C levels and non-HDL-C levels vs. the placebo group at week 56 [217]. Another study designed to evaluate the long-term efficacy of phentermine/topiramate treatment found that the HDL-C levels of study participants increased more than in the placebo group [218].

Orlistat is an intestinal lipase inhibitor that prevents breakdown of triglycerides and has an excellent long-term safety record [216]. Interestingly, orlistat causes a 25% reduction in cholesterol absorption [219]. Regarding orlistat-induced changes in HDL-C levels, studies are inconsistent. Some studies reported a significant increase of HDL-C in patients receiving orlistat [200,201,202,203,204,205,206,207,208,209,210,211,212,213,214,215,216,217,218,219,220,221,222], while others observed no significant changes [223,224,225].

Noteworthily, food intake only minimally affects HDL-C [226,227], which might explain the inconsistent effects of orlistat on HDL-C levels.

Lorcaserin is a serotonin 2c receptor agonist available in the USA that increases central serotonin release and has been shown to be effective for long-term weight management [228,229]. A recent study showed that lorcaserin treatment for six months resulted in decrease of LDL-C, while plasma levels of HDL-C were increased [230]. Lipid subfraction analysis further revealed an increase in HDL particle size.

Liraglutide is a glucagon-like peptide-1 receptor agonist widely used to treat type 2 diabetes. This drug further increases satiety, slows gastric emptying, and also decreases body weight, besides reducing glucose concentration [231]. Long-term treatments with liraglutide have been shown to reduce body weight and waist circumference, but also to improve plasma lipid levels, including an increase in HDL-C levels [232,233].

Overall, most pharmacological approaches for obesity treatment increase HDL-C. Further studies examining potential effects of anti-obesity treatment on metrics of HDL function are warranted.

## 8. Effects of Dietary Approaches on HDL Levels and Function

Other strategies to treat obesity, besides pharmacological treatments and surgical procedures, are hypocaloric diets, such as intermittent fasting and caloric restriction. Furthermore, dietary patterns including Mediterranean diet are commonly used to induce weight loss and improve cardiovascular health in obese individuals [234,235].

Caloric restriction is the most common form of dietary restriction, in which subjects strive to decrease their daily energy intake by 15–40% of baseline needs each day [236]. In a 16-week intervention trial in which obese diabetic participants were given a very low calorie diet (450 kcal/day), caloric restriction was shown to reduce CETP activity and increase ApoA-I levels, but did not affect HDL-C levels or HDL cholesterol efflux capacity [237]. Another recently published study compared the effect of an 8-week intermittent caloric restriction regimen to continuous caloric restriction in overweight and obese subjects. They observed that these interventions similarly reduced body weight and fat mass and improved plasma triglycerides but had no effect on levels of HDL-C [238]. Interestingly, Liang et al. observed that a 3-month intervention of caloric restriction, together with moderate physical activity, resulted in weight reduction in obese subjects with metabolic syndrome but decreased PON1 levels [239]. In line with this, another study with obese participants observed that a low-calorie diet reduced PON1 enzyme activity [240]. Furthermore, weight loss through caloric restriction has been shown to decrease LCAT activity in obese [241] as well as in normal-weight subjects [242].

Alternate-day fasting (ADF) regimens consist of a “feeding day”, with ad libitum feeding and a “fasting day”, with complete abstinence of food and drink intake, except for water for 24 h. These regimens are less common than caloric restriction but were created to facilitate compliance with dietary restriction protocol, as these regimens require energy restriction only every-other day. In a modified ADF study, in which obese participants were allowed to consume 25% of their regular energy needs on the fasting day, body weight and body fat decreased and also levels of triglycerides, total cholesterol, and LDL-C decreased, whereas levels of HDL-C remained unchanged [243]. Varady et al. demonstrated that the same ADF regimen was effective in both weight reduction and cardioprotection in normal-weight and overweight subjects [244]. After 12 weeks of ADF, the study participants showed decreased body weight and fat mass, but no changes in the levels of HDL-C were observed. Similar results were observed in another ADF intervention study in normal-weight participants [245].

Mediterranean diet is a dietary approach to induce weight loss and to prevent cardiovascular events [234]. This diet pattern is generally characterized by high consumption of vegetables, fruits, nuts, legumes, wheat-based cereals, olive oil, and fish; moderate consumption of dairy products and poultry; and low consumption of red and processed meats [246]. In the Prevention with Mediterranean Diet study (PREDIMED), individuals with high cardiovascular risk were assigned to a Mediterranean diet supplemented with extra-virgin olive oil or nuts and had lower incidence of cardiovascular events than the control group, assigned to a reduced-fat diet [247]. A substudy, including volunteers of the PREDIMED trial, concentrated on examining the effect of this anti-oxidant-rich dietary pattern on HDL function. Of particular interest, they observed that a 1-year Mediterranean diet, enriched with olive oil or nuts, increased the HDL cholesterol efflux capacity, PON1 activity, and HDL vasodilatory activity [248]. Similarly, another study showed that 12 weeks of Mediterranean diet and exercise improved HDL cholesterol efflux capacity and improved HDL function by inhibiting myeloperoxidase-mediated oxidative stress in subjects with metabolic syndrome [249].

## 9. Conclusions

Obesity leads to a depletion of HDL-C, due to a marked shift from large cholesteryl-ester-rich HDL to small and dense triglyceride-rich particles. The mechanisms underlying this shift are multifactorial, including elevated CETP activity linked to increased levels of triglyceride-rich lipoproteins, lower adiponectin levels, and increased clearance of large HDL particles. These changes in HDL subspecies are accompanied by changes in composition and functionality. S1P will potentially be attached to alternative chaperones, resulting in attenuated multiple beneficial effects of S1P. Bariatric surgery is currently the most effective treatment for raising HDL-C levels and, more importantly, it also significantly improves HDL functionality and may be related, at least in part, to the reduction in mortality observed in observational studies. In addition, there is accumulating evidence that Mediterranean diet, especially when enriched with virgin olive oil, significantly enhances parameters of HDL atheroprotective functions. Further studies are warranted to identify specific components in olive oil or other nutrients that improve HDL function. Most pharmacological approaches for obesity treatment increase HDL-C but further studies examining potential effects of anti-obesity treatment on metrics of HDL function are needed. The data of caloric restriction strategies are inconsistent and even show negative effects on some metrics of HDL functionality.

Considerable interest has recently focused on approaches to influence the biological functions of HDL in the search for new cardioprotective therapies and might establish novel treatment strategies in obese individuals.

## Figures and Tables

**Table 1:** Representation of HDL heterogeneity.

HDL Subclass	Size	Shape	Abundant Components	Important Functions
Pre-β HDL	9.6 nm diameter, 4.7 nm thickness	discoidal	ApoA-I, phospholipids	ABCA1-Cholesterol efflux
HDL3	7.5 nm, 175 kDa	spherical	Protein:lipid ratio 55:45 PON1, ApoA-II, ApoM, S1P	Anti-oxidative activity Anti-inflammatory activity ABCA1-Cholesterol efflux
HDL2	10 nm, 350 kDa	spherical	Protein:lipid ratio 40:60	ABCG1- Cholesterol Efflux

Apo, apolipoprotein; ABCA1, ATP-binding cassette transporter A1; PON1, paraoxonase 1; ABCG1, ATP-binding cassette subfamily G member 1.
